# Are Graduate Students Rational? Evidence from the Market for Biomedical Scientists

**DOI:** 10.1371/journal.pone.0082759

**Published:** 2013-12-23

**Authors:** Margaret E. Blume-Kohout, John W. Clack

**Affiliations:** Robert Wood Johnson Foundation Center for Health Policy and Department of Economics, University of New Mexico, Albuquerque, New Mexico, United States of America; University College London, United Kingdom

## Abstract

The U.S. National Institutes of Health (NIH) budget expansion from 1998 through 2003 increased demand for biomedical research, raising relative wages and total employment in the market for biomedical scientists. However, because research doctorates in biomedical sciences can often take six years or more to complete, the full labor supply response to such changes in market conditions is not immediate, but rather is observed over a period of several years. Economic rational expectations models assume that prospective students anticipate these future changes, and also that students take into account the opportunity costs of their pursuing graduate training. Prior empirical research on student enrollment and degree completions in science and engineering (S&E) fields indicates that “cobweb” expectations prevail: that is, at least in theory, prospective graduate students respond to contemporaneous changes in market wages and employment, but do not forecast further changes that will arise by the time they complete their degrees and enter the labor market. In this article, we analyze time-series data on wages and employment of biomedical scientists versus alternative careers, on completions of S&E bachelor's degrees and biomedical sciences PhDs, and on research expenditures funded both by NIH and by biopharmaceutical firms, to examine the responsiveness of the biomedical sciences labor supply to changes in market conditions. Consistent with previous studies, we find that enrollments and completions in biomedical sciences PhD programs are responsive to market conditions at the time of students' enrollment. More striking, however, is the close correspondence between graduate student enrollments and completions, and changes in availability of NIH-funded traineeships, fellowships, and research assistantships.

## Introduction

**Figure 1:**
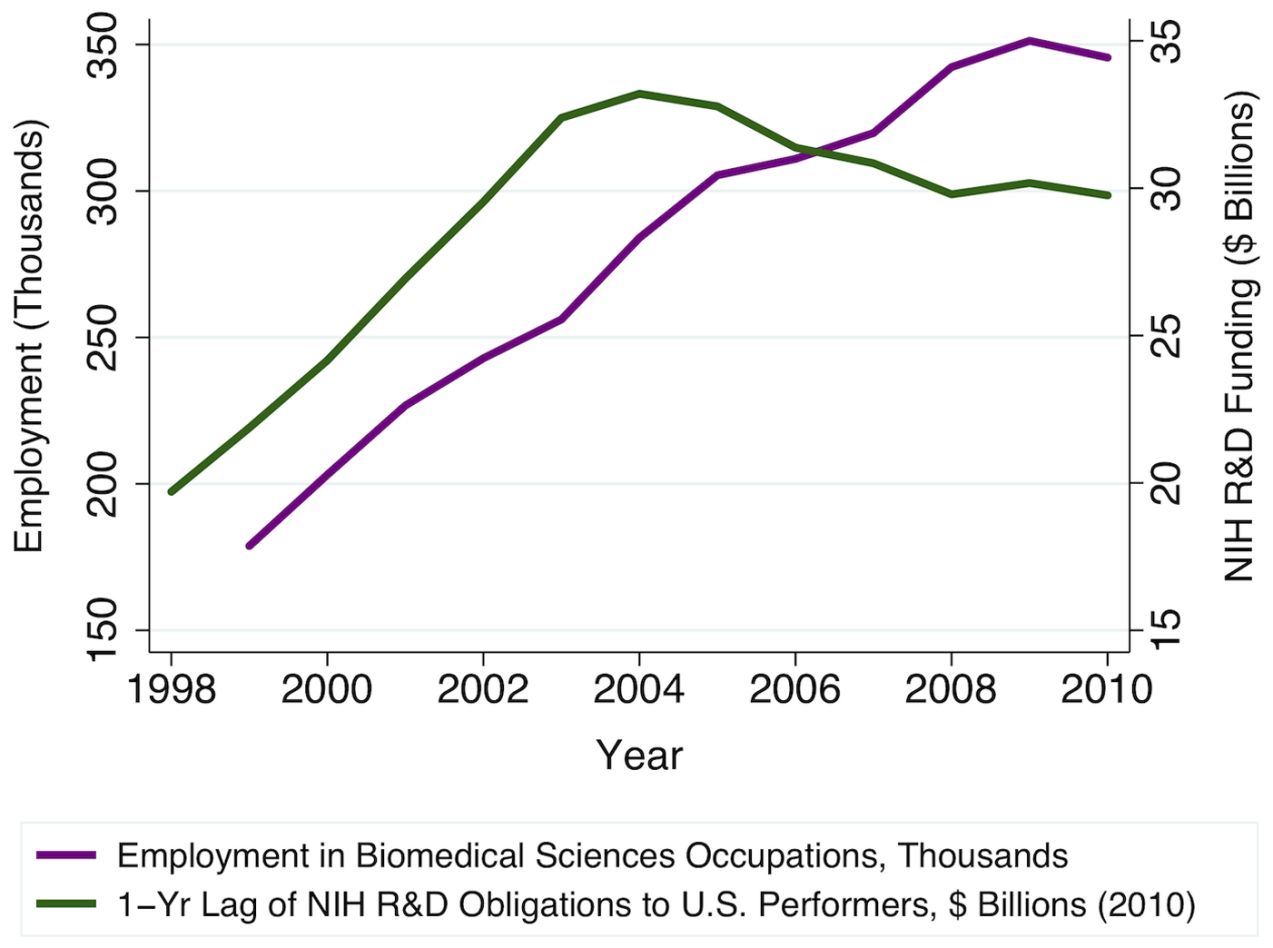
Changes in employment mirror NIH R&D obligations during the NIH doubling period. Estimated employment counts obtained from U.S. Bureau of Labor Statistics Occupational Employment Statistics for biomedical sciences occupations, and one-year lagged U.S. National Institutes of Health (NIH) research and development (R&D) obligations to U.S. performers, in billions of constant 2010 dollars (converted using the Biomedical Research and Development Price Index (BRDPI)), presented for years 1998 through 2010.

**Figure 2:**
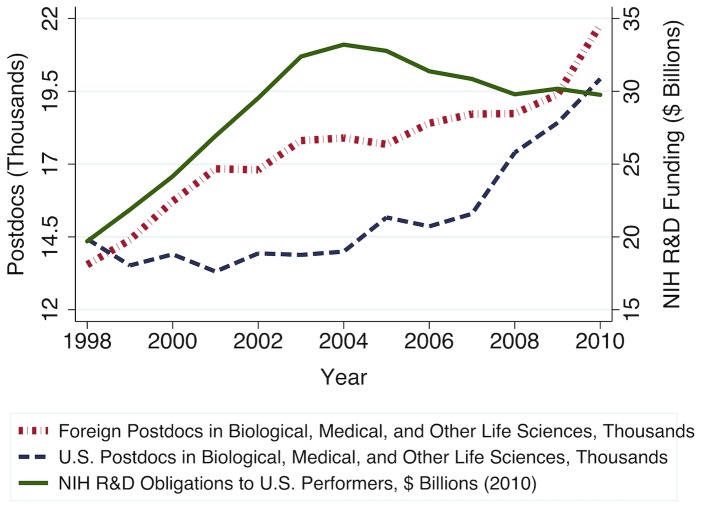
Increase in demand for biomedical sciences postdocs was largely met by foreign temporary residents. U.S. National Institutes of Health (NIH) R&D obligations to U.S. performers, in billions, from the National Science Foundation (NSF) Survey of Federal Funds for Research and Development, inflated to constant 2010 dollars using the Biomedical Research and Development Price Index (BRDPI), for years 1998 through 2010. Estimated counts of U.S. citizen and permanent resident postdoctoral researchers versus foreign temporary resident postdoctoral researchers in biomedical sciences and related fields departments and programs, in thousands, calculated using data from the NSF-NIH Survey of Graduate Students & Postdoctorates in Science and Engineering.

U.S. Congressional appropriations for the National Institutes of Health (NIH) grew at an unprecedented rate in the last decade, increasing from approximately $13.7 billion in fiscal year 1998 to almost $28.1 billion by fiscal year 2004. This increase in public research funding substantially increased total demand for biomedical sciences research, which in turn increased employment in biomedical sciences occupations (Fig 1). Garrison et al. (2005) note that, due to the production lag inherent to PhD training, the rapid increase in postdoctoral researchers they observed during this period was largely attributable to an influx of foreign-trained PhDs, many of whom came to the U.S. to fill temporary research positions (Fig 2) [1]. However, this observed increase in postdocs – positions that have become near-ubiquitous as a career waypoint for freshly minted PhDs – was not accompanied by an increase in more permanent faculty positions [2]. For example, even among elite NIH National Research Service Awardees who began their postdoctoral fellowships in 1992–1994, after eight years only 27% were in tenured or tenure-track positions [3].

Enrollments in U.S. biomedical sciences graduate programs also increased during the NIH budget doubling period, yielding a lagged pulse of new PhDs who completed their degrees and entered the job market just as NIH funding levels stagnated. Labor market effects of the rapid growth and subsequent real decline in NIH funding were likely further exacerbated by the Great Recession: a result of counter-cyclical enrollment in graduate programs and senior faculty postponing retirement [4]. Since 2006, the total number of PhDs produced each year by U.S. biomedical sciences graduate programs has generally exceeded growth in related jobs. This apparent oversupply of biosciences PhDs has prolonged the typical postdoctoral training period, and has lowered PhDs' relative wages at career entry. These developments have generated concern among academics, practitioners, and policymakers about how best to support and sustain the nation's biomedical sciences research workforce [5], [6], [7], [8]. In her recent book, *How Economics Shapes Science*, Paula Stephan (2012) summarizes these trends:

Job prospects have been particularly dismal in the biomedical sciences. But still students continue to enroll in PhD programs. Many are foreign born, but some are U.S. born. Why? Why, given such bleak job prospects, do people continue to come to graduate school? [9].

Even though new life sciences PhDs have relatively high rates of employment, in recent years many graduates have reported taking jobs outside of scientific research, of which many do not require or directly utilize their doctoral training. This disconnect between students' human capital investment decisions and their ultimate occupational outcomes has prompted many observers to argue that prospective graduate students are poorly informed with respect to their future job prospects [2], [3], [9].

In this article, we examine whether job market signals influence graduate students' enrollment decisions. In theory, prospective graduate students should make their enrollment decisions based on a full understanding of their expected future career prospects, including various careers' inherent non-pecuniary benefits. Sociologists and education researchers have documented many non-financial influences that may affect a given individual's decision to enter and complete a PhD program, including parental educational attainment, characteristics of the prospective student's undergraduate institution, undergraduate debt accumulation, racial stigma and gender marginalization, and early access to advanced college-preparatory coursework [9], [10], [11], [12], [13]. Once students enter, their decision to remain and complete the PhD and ultimately pursue a career in scientific research, in academia or otherwise, likewise may be affected not only by perceived availability of jobs, but also other attributes of prospective careers such as the intellectual challenge, job security, availability of research funding, opportunities to collaborate, and so on [14], [15]. A recent review of various explanations offered for underrepresentation of women and minorities in S&E fields provides little mention of differences in the labor market incentives that prospective students face [16]. However, one study did document gender bias in salaries, teaching loads, administrative support, and scholarly recognition among academic chemists [17]. If female undergraduates increasingly perceive such disparities among the faculty, such career-related concerns may discourage rational young women from pursuing PhDs even when the expected financial returns are improving.

Likewise, changes in federal policies – like those studied in this article – may also affect students' decision-making indirectly, above and beyond the effects of policy changes on wage price signals and number of scientists employed. For example, faculty members' frustration and uncertainty due to the increased competition and administrative burdens for research grant funding can affect the training climate, and encourage students to opt for alternative careers [5]. Student concerns such as these may also help to explain Sauermann and Roach's (2012) observation that 39% of students in life sciences rate research-focused faculty positions as “extremely attractive” while still early in their PhD program, but that fraction drops to 33% as they approach graduation [18].

Even so, if we assume that these non-financial incentives and barriers remained relatively constant during our analytic period (1998–2010), then in theory the recent decline in jobs growth and relatively low starting salaries for entry-level PhD researchers should have dampened prospective students' enthusiasm for pursuing graduate training [7], [9]. Below, we discuss the theoretical framework in more detail, then present our empirical results.

Rational expectations models have been used in macroeconomics since the 1970s to help explain persistent disequilibria in relationships between prices (including wages), output, and employment [19]. Though the details of these models may differ, rational expectations models commonly assume that agents anticipate and respond to future market conditions. These anticipated changes, furthermore, include changes not only in the market price for an individual's own output, but also those changes relative to other price changes throughout the economy. For prospective graduate students considering investing in their human capital, the rational expectations model implies they should not (and do not) consider contemporaneous changes in market wages for biomedical scientists. That is, their decision to enter a PhD program in a given year should not be based on changes that same year in wages or employment among scientists who already hold PhDs. Instead, prospective students should anticipate future labor market and macroeconomic conditions, accounting for responses of already-trained scientists and other prospective students, since by assumption these individuals share the same information set and expectations for the future. Finally, prospective students should compare the benefits of earning a PhD and pursuing a career in biomedical sciences versus the benefits of their next best alternative.

One difficulty with applying these models is that they presume agents (in our case, prospective graduate students) have sufficient information from experience to make rational forecasts of the future. As Ryoo and Rosen (2004) discuss, this assumption does not seem very realistic for young adults who are considering whether to enter professional labor markets [20]. To address this concern, the NIH Advisory Committee to the Director (ACD) Biomedical Research Workforce working group recommended that faculty and institutions provide students with better information about their graduate students' placement and expected career trajectories [8]. Yet, with median time-to-degree for biomedical sciences PhDs exceeding five years, even prospective students who are relatively well-informed with respect to past and current market conditions may still fail to anticipate future changes in the biomedical PhD market. This imperfect foresight is hardly unique to biomedical sciences PhDs: Freeman (1976) and others have previously observed that highly skilled occupations are subject to a substantial “production lag,” with labor supply largely predetermined by students' entry into training programs several years prior [21].

To understand how market signals impact the labor supply and human capital investment decisions of current and prospective biomedical scientists, we need also to understand how changes in NIH funding levels affect both the demand for biomedical scientists' labor and its supply. The current structure of the U.S. biomedical research enterprise relies heavily on trainees – graduate students and postdoctoral researchers – to perform lab work [7], [9]. As NIH extramural grant funding at U.S. universities increased during the budget doubling period, faculty seeking to staff their laboratories not only hired more postdocs (Fig 2), but also hired more graduate research assistants (GRAs). Blume-Kohout and Adhikari (2012) find that increases in universities' life sciences R&D funding during this period yielded approximately proportional increases in graduate research assistantships [22]. Although such increases in external sources of support might also have encouraged some universities to reallocate internal institutional funding, thus benefitting other departments and programs [23], Blume-Kohout and Adhikari show that increases in availability of NIH research assistantships within biomedical sciences programs do typically yield increases in total enrollment for those same programs.

For our empirical analysis, we first extracted microdata from several nationally representative surveys to calculate statistical time series variables. For example, we used these microdata to estimate average wages among individuals in biomedical sciences occupations, by year. We then combined these statistical time series with annual macroeconomic time series data, such as total NIH funding and Gross Domestic Product. These combined data enable us to evaluate the relative importance of changing labor market conditions, as well as the particular (and plural) roles of NIH funding, in inducing students towards careers in biomedical sciences. Other variables in our analytic time series dataset – all of these organized with the year as the unit of observation – include first-time graduate student enrollments and PhD completions, the share of students funded on NIH research assistantships, traineeships and fellowships, estimates of biomedical scientists' and alternative occupations' salaries and employment rates, and NIH and biopharmaceutical industry R&D expenditures. In light of the Great Recession and related potential for countercyclical enrollment [4], we explicitly control for relative attractiveness of prospective graduate students' alternative career options. We also compare the magnitudes of the employment responses associated with an increase in federal R&D funding versus an increase in biopharmaceutical industry R&D expenditures, and investigate the dual role of NIH R&D funding in stimulating both demand and supply in the market for biomedical scientists. Finally, to separate out possible simultaneous effects of changes in NIH funding levels on pre-doctoral and postdoctoral labor markets and to permit causal inference, we employ instrumental variables econometric estimation [24].

**Figure 3:**
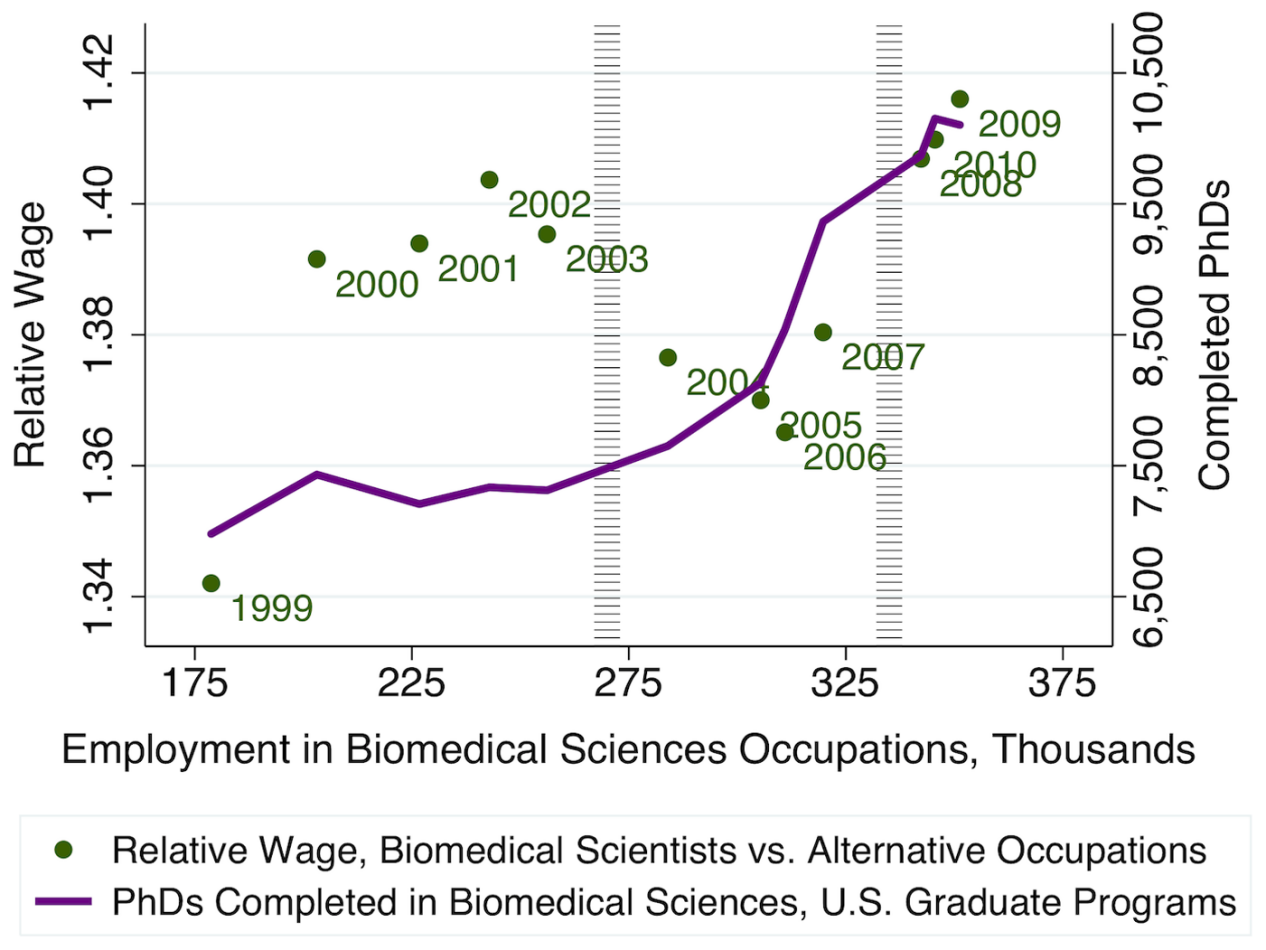
Training lags for new PhDs yielded a short-run increase in biomedical scientists' relative wages. Relative wage calculated as the ratio of the employment-weighted average wage for biomedical sciences occupations, versus the employment-weighted average wage across other occupations held by individuals with bachelor's degrees in biological sciences and chemistry-related fields, using American Community Survey (ACS) 2009 field-of-degree and occupation microdata and U.S. Bureau of Labor Statistics Occupational Employment Statistics (BLS OES) estimates of average wages by occupation, for years 1999 through 2010. Plot line shows the correlation between total PhDs completed in U.S. biomedical sciences programs by year, estimated using microdata from the National Science Foundation's Survey of Earned Doctorates/Doctorate Records File (SED/DRF data), with biomedical sciences occupations' annual employment estimates from BLS OES data.

**Figure 4:**
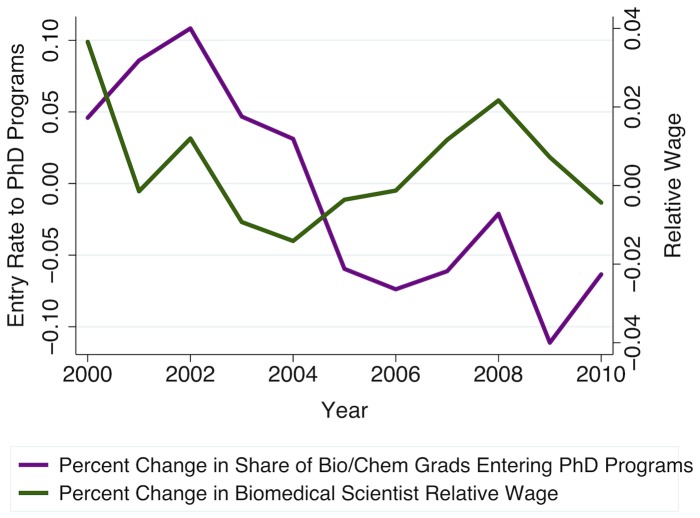
Graduate student enrollments respond to current changes in relative wages. Relative wages calculated here as in Figure 3, then logged and first-differenced to estimate year-to-year percentage changes. Relative entry to PhD programs is calculated as the total number of first-time, full-time graduate students enrolling in U.S. biomedical sciences graduate programs, estimated from NIH-NSF Survey of Graduate Students & Postdoctorates in Science and Engineering (GSS) microdata, versus the number of bachelor's degrees awarded by U.S. institutions in biological sciences and chemistry-related fields, as provided by the U.S. Department of Education's Integrated Postsecondary Education Data System (IPEDS). Relative entry is also log-transformed and first-differenced, to allow interpretation as percentage change in enrollments versus those the previous year.

**Figure 5:**
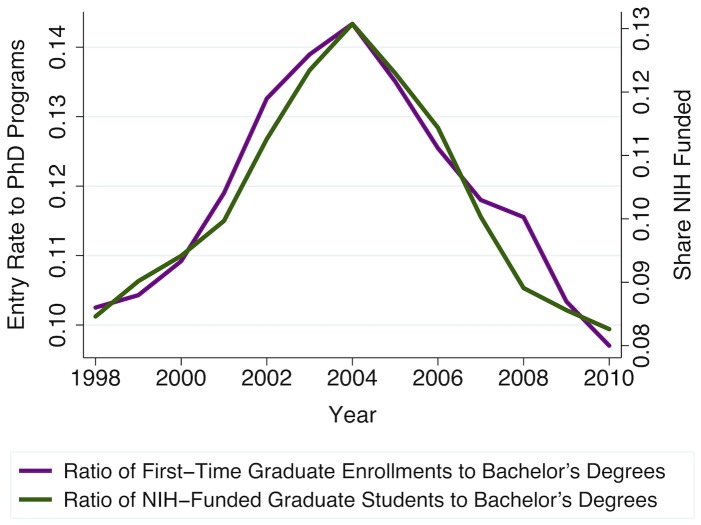
Biosciences graduate student enrollments are very sensitive to changes in NIH support. Entry rate to PhD programs is the relative entry ratio defined in Figure 4. Counts of NIH-supported full-time graduate students in U.S. biomedical sciences departments and programs are also derived from the NIH-NSF Survey of Graduate Students & Postdoctorates in Science and Engineering. Share NIH Funded is the ratio of new enrollments, thus calculated, to the total number of bachelor's degrees awarded in biological sciences and chemistry-related fields each year, estimated using IPEDS data, also as in Figure 4.

As illustrated in Figures 1 and 3, the NIH budget doubling period (1998–2003) was associated with an increase in demand for biomedical sciences research, which, in turn, induced derived demand for biomedical scientists, resulting both in higher relative wages and in increased employment in biomedical sciences occupations. Consistent with the cobweb expectations model, we observe that short-run increases in biomedical scientists' relative wages appear to encourage contemporaneous increases in first-time, full-time graduate student enrollments (Fig 4). However, Figure 5 shows that these enrollments track even more closely with current availability of NIH funding for students. Since the NIH budget expansion impacted NIH-funded graduate research assistantships, jobs for PhD biomedical scientists, and biomedical scientists' relative wages, we turn to econometric analysis to identify and compare the relative importance of each of these influences on prospective graduate students' decisions to enroll. We find that enrollment in biomedical sciences graduate programs is highly responsive to current fluctuations in biomedical scientists' relative wages, such that a 1% increase in current wages is associated with about a 3.4% increase in graduate students' enrollment. However, availability of NIH funding for students is also highly significant, with each additional NIH-funded traineeship, fellowship, or research assistantship increasing new (first-time, full-time) enrollments by, on average, one additional student that same year.

**Figure 6:**
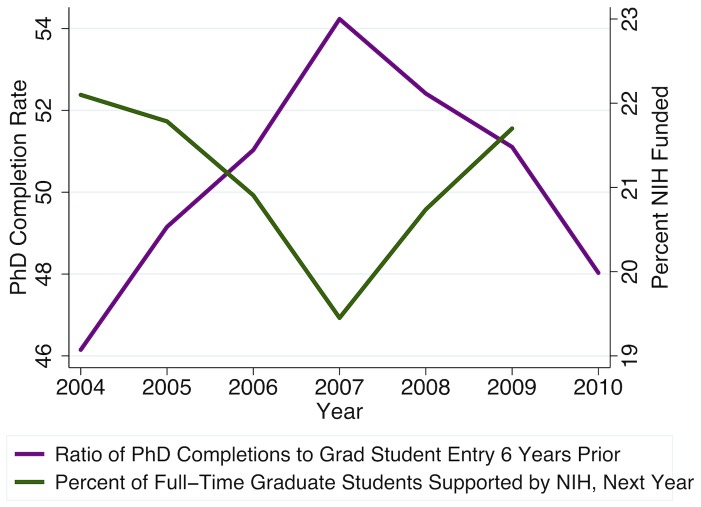
Six-year PhD completions decline when a higher percentage of students have NIH funding. PhD completions rate calculated as the ratio of biomedical sciences research doctorates awarded (SED/DRF data, see Figure 3) to the number of first-time full-time graduate students enrolling in PhD-granting biomedical sciences programs six years prior (GSS data, see Figure 4). Percent NIH funded is the share of full-time U.S. biomedical sciences graduate students with any NIH support (research assistants, trainees, fellows, and other mechanisms), also from GSS, for the following year, representing the alternative to PhD students completing their degree and going on the job market.

Finally, we considered effects of NIH support on timely PhD completions. As shown in Figure 6, when a higher percentage of graduate students have NIH support, a lower percentage of those enrolled graduate with PhDs within six years. Our econometric results indicate this represents a combination of opposing effects. On the one hand, when more research assistantship funding is available, additional students may be admitted and choose to enroll, but unless the high-quality applicant pool also expands, these marginal (i.e., additional) students may be more likely to drop out, or otherwise fail to complete a PhD. On the other hand, advanced students in dissertation stage may be more likely to delay their graduation when NIH-funded traineeships, fellowships, or research assistantships are available for their support. Finally, since increases in NIH R&D obligations are associated with higher demand for biomedical scientists as an input to research production, if relative wages and employment opportunities increase while a student is in school, then we may also observe lower rates of PhD completion relative to enrollment six years prior, due to an increase in the number of students leaving with only Master's degrees.

## Empirical Approach

### Data

Our analytic time-series dataset includes variables constructed from several different data sources. Summary statistics for key analytic variables are presented in Table 1, and their data sources and details of their construction are provided below.

**Table 1:** Descriptive Statistics for Constructed Variables, 1999–2010.

Variable	Mean	St. Dev.
Employment in Biomedical Sciences Occupations	280,568	58,300
Total Employment in All Other S&E Occupations	120,912,380	3,242,727
Average Salary for Biomedical Sciences Occupations, $2010	$74,667	$4,057
Weighted Average Salary, Alternative Occupations of Biology & Chemistry Bachelor's degree holders, $2010	$53,790	$2,360
Biopharmaceutical Industry R&D Expenditures, $2010 Billions, PhRMA Member Companies estimates	$35.8	$3.97
Biopharmaceutical Industry R&D Expenditures, $2010 Billions, Bureau of Economic Analysis estimates	$51.2	$15.7
US National Institutes of Health R&D Obligations to Domestic Performers, $2010 Billions	$29.4	$3.46
Earned Bachelor's Degrees, Biological Sciences & Chemistry	172,465	32,185
First-Time, Full-Time Graduate Enrollments in Biomedical Sciences	20,416	2,528
Full-Time, NIH-Supported Biomedical Sciences Graduate Students	17,569	2,172
Total Full-Time Biomedical Sciences Graduate Students	82,219	10,226
Completed PhDs in Biomedical Sciences Fields	8,340	1,220

#### Bureau of Labor Statistics Occupational Employment Statistics

Salary estimates for biomedical scientists and alternative career fields were calculated for years 1999 through 2010 using data from the U.S. Department of Labor Bureau of Labor Statistics' (BLS) Occupational Employment Statistics (OES) survey [25]. OES data are collected semiannually from 200,000 business establishments, and the wage estimates reported each year are produced by combining data from the current period's survey with data with data collected in the previous two surveys. For example, estimates reported for May 2012 are calculated using data from the May 2012, November 2011, and May 2011 surveys. Although wage data are available from BLS for 1997 onwards, in 1999 BLS switched from its own occupational classification system to use the Office of Management and Budget's Standard Occupational Classification (SOC) codes. As BLS observed, many of the old BLS occupation codes did not map one-to-one to SOCs [26]. Due to these data constraints, our analytic time series is limited to the twelve-year period noted above.

To generate the biomedical scientist wage series, we used the occupations identified in the recent NIH Advisory Committee to the Director (ACD) task force report [8]. These occupations and their respective SOCs are listed in Table 2. Our wage estimate for each year is constructed as a weighted average, in which the weights for each occupation code are determined by the number of people employed in that occupation code, relative to the sum total of employment across all biomedical sciences occupations. To represent the opportunity cost associated with a pursuing career in biomedical sciences, we constructed two alternative career wage series estimates using OES data. The first series is the weighted average salary for individuals holding bachelor's degrees in any S&E field, as identified in the ACS data. The second series includes only those individuals who earned bachelor's degrees in biological sciences or chemistry-related fields, and who have not earned (and are not currently earning) a graduate degree. We constructed this second series by combining ACS data with data from the Survey of Doctorate Recipients (SDR), also described below.

**Table 2:** Biomedical Scientist Occupation Codes for BLS OES Data.

Occupation Code	Occupation Title
11–9121	Natural Sciences Managers
17–2031	Biomedical Engineers
19–1021	Biochemists and Biophysicists
19–1022	Microbiologists
19–1023	Zoologists and Wildlife Biologists
19–1041	Epidemiologists
19–1042	Medical Scientists, except Epidemiologists
19–4021	Biological Technicians
25–1042	Postsecondary Biological Sciences Teachers

Finally, we used OES counts of the number of people employed in biomedical sciences occupations versus all other occupations chosen by individuals with bachelor's degrees in S&E fields to estimate *relative* growth in biomedical scientists' employment over time.

### National Science Foundation Survey of Doctorate Recipients (SDR)

The SDR is a longitudinal survey targeting individuals who received research doctorates in S&E- and health-related fields from U.S. institutions. Collected by the U.S. National Science Foundation (NSF) every two to three years, the SDR follows a sample of individuals from the time they receive their PhD until they reach age 75.

For this article, we used publicly-available SDR data to identify bachelor's degree fields that are most strongly associated with students going on to earn PhDs in life sciences fields. Reviewing survey data collected from 1999 through 2008, we found that more than 80% of life sciences PhDs had earned their bachelor's degrees in fields related to biological sciences and chemistry, consistent with earlier literature discussing the linear progression of study in science [27]. Although health-related majors such as nursing were also represented, a much smaller fraction of individuals in those majors chose to pursue research PhDs. As such, we felt that the distribution of alternative occupations chosen by biological sciences and chemistry majors would be most representative of the other opportunities prospective PhD students might consider. With this in mind, we turned next to the 2009 American Community Survey.

### American Community Survey (ACS) Public Use Microdata Sample

We began by identifying all respondents in the 2009 ACS Public Use Microdata Sample (PUMS) who had earned bachelor's degrees in biological sciences and chemistry-related fields. The specific ACS field-of-degree codes we used are listed in Table 3. Then, we identified all SOC codes associated with those degrees, and calculated the survey-weighted share of all biological sciences and chemistry bachelor's-degree holders for each 4-digit SOC. Finally, we merged these calculated shares from ACS with BLS OES wage data on SOC, and used these shares to estimate the weighted average salary by year for bachelor's degree holders in these alternative occupations.

**Table 3:** American Community Survey undergraduate field-of-degree codes for biological sciences and chemistry-related fields.

Code	Field of Degree
*Biological Sciences Fields*	
3600	Biology
3601	Biochemical Sciences
3602	Botany
3603	Molecular Biology
3604	Ecology
3605	Genetics
3606	Microbiology
3607	Pharmacology
3608	Physiology
3609	Zoology
3611	Neuroscience
3699	Miscellaneous Biology and Epidemiology
2402	Biological Engineering
2404	Biomedical Engineering
4002	Nutritional Sciences
5102	Applied Biotechnology
*Chemistry Fields*	
5003	Chemistry
2405	Chemical Engineering

### NSF-NIH Survey of Graduate Students and Postdoctorates in Science and Engineering

This survey collects data on part- and full-time graduate student enrollments and postdoctoral hires by race, gender, and citizenship, as well as full-time graduate students' primary source of financial support, reported by S&E degree-granting departments and programs at U.S. academic institutions. For this analysis, we extracted and summed annual counts of postdocs by citizenship (used in Fig 2), first-time full-time graduate students, total enrollments, and full-time graduate students whose primary source of financial support is the NIH (including NIH-funded research assistantships, traineeships, or fellowships) across academic programs that grant PhDs in biological, medical, and other life sciences. We use these data to construct our annual estimates of the proportion of graduate students with NIH funding, calculated as the number of full-time biomedical sciences graduate students with NIH support, *F_t_*, divided by the total full-time graduate enrollment in those programs, *S_t_*.

### Integrated Postsecondary Education Data System (IPEDS) Completions

The National Center for Education Statistics' IPEDS Completions survey provides our counts of students completing bachelor's degrees in biological sciences and chemistry-related fields each year at U.S. institutions. These census data are collected each Spring from all U.S. institutions of higher education that participate in Federal student financial aid programs. Combined with the graduate enrollments data described above, these counts permit us to control for exogenous year-to-year changes, for example due to demographic trends or general macroeconomic conditions, that could impact the number of domestic students earning their bachelor's degrees in relevant fields each year. Specifically, we use these data to estimate *relative* entry into graduate programs, as the ratio of first-time full-time students entering U.S. biomedical sciences graduate programs each Fall, *G_t_*, to bachelor's degrees awarded in biological sciences and chemistry the previous Spring, *B_t_*. As shown in Figure 5, we find that relative enrollments in biomedical sciences graduate programs are strongly correlated with availability of NIH funding for graduate students.

### NCSES-NSF Survey of Earned Doctorates/Doctorate Records File (DRF)

The National Center for Science and Engineering Statistics (NCSES) at the NSF oversees data collection on the complete population of students graduating with PhDs each year at U.S. institutions, via its annual Survey of Earned Doctorates. The DRF contains survey responses for each year since the survey's inception in 1957. We use publicly-available DRF data to determine the number of PhD completions in biomedical sciences fields each year. We then construct our proxy variable for six-year PhD completions as the ratio of PhDs completed to graduate student enrollments six years prior, to control for differences across years in PhD completions that are simply due to prior changes in first-time graduate student enrollments. The direct and indirect effects of changes in NIH funding on six-year PhD completion rates are of specific policy interest, as the NIH ACD report includes a recommendation that PhD students' funding support be capped at six years [8]. However, our relative PhD completions variable includes all first-time, full-time graduate students entering the programs six years prior in the denominator, which – if our aim were to calculate the PhD programs' actual completion rates – would introduce measurement error due to its inclusion of students that only intend to pursue a Master's degree, as well as its exclusion of part-time PhD students. Although we do, as discussed above, restrict our enrollment counts to programs offering research doctorates in biomedical sciences and related fields, some of these programs may also offer terminal Master's programs to which they admit full-time students. In addition, whether overtly or not, some students may enter PhD programs with no intention of earning a PhD, expecting instead to leave with only a Master's degree. In either case, our six-year PhD completions rate may be lower than the “true” rate of completion for PhD programs, due to inclusion of some Master's-only students in the denominator. However, since our empirical analyses consider year-to-year *changes* in this rate, as opposed to its absolute level, this measurement error would only impact our results if, over our period of study, there was a substantial and disproportionate change in admissions and enrollment for standalone Master's degree programs, and furthermore the change in proportion of Master's-only enrollments was positively correlated with NIH-funded graduate student support. Otherwise – if there is no significant change in proportion or if the correlation between Master's program enrollment and NIH funding support is negative – this measurement error would bias our estimated effect of NIH funding towards zero, potentially causing us to perceive no statistically significant effect of NIH funding on PhD completions.

Like Figure 5, Figure 6 demonstrates graphically the strong dependence of full-time graduate student enrollment on relative availability of NIH funding. However, Figure 6 also indicates that the six-year PhD completion rate rises when NIH support for students contemporaneously declines, which suggests when NIH-funded support mechanisms become less readily available, current students may be more motivated to complete their degrees in a timely fashion.

### Macroeconomic Data Series

In addition to the survey microdata described above, we also employ four macro-level data series to investigate and control for effects of exogenous shifts to market demand. Total NIH R&D obligations to U.S. performers for each year were obtained from the NSF Survey of Federal Funds for R&D, adjusted to constant 2010 dollars using the Biomedical Research and Development Price Index (BRDPI). We calculate Real U.S. Gross Domestic Product (GDP) in chained 2010 dollars with U.S. Bureau of Economic Analysis (BEA). We then calculate relative NIH R&D funding as the ratio of NIH obligations to U.S. performers over U.S. GDP.

Finally, we include two alternative measures of annual biopharmaceutical industry R&D expenditures in the U.S., both of which are likewise divided by U.S. GDP. The first is Pharmaceutical Research and Manufacturers of America (PhRMA) trade association members' reported expenditures on domestic R&D, adjusted to constant 2010 dollars using the BRDPI. This series includes all U.S. domestic R&D expenditures by the association's members, including both U.S.- and foreign-owned firms. The second variable uses BEA R&D satellite accounting data for pharmaceutical and medicine manufacturers (NAICS code 3254) reported for years 1998 through 2007, and extrapolates that series through 2010 as a function of annual changes in PhRMA-reported expenditures [28]. In contrast with the PhRMA series, the BEA data excludes foreign-owned firms but includes all U.S. pharmaceutical industry-performed and industry-funded R&D. These two measures of industry R&D expenditures are particularly important to our analysis, enabling us to estimate effects of wages on labor supply and employment levels separately and consistently, as discussed in section II.B. below.

### Econometric Models

Markets for highly-skilled labor are subject to substantial production lag, with labor supply largely predetermined by students' entry into training programs several years prior [21]. We compared empirical results from a variety of models, in order to assess whether prospective PhD students appear to forecast future job market conditions when deciding to enroll, as in the rational expectations model, or alternatively whether they exhibit cobweb expectations.

We estimate the cobweb expectations models empirically by assuming that present-day, time *t* enrollment is determined by present-day, time *t* market conditions. In essence, these models assume participants in the biomedical sciences labor market do have information about current market conditions, but they are committing to a future labor market for which they may have no better indication of wage rate than the present wage. In contrast, our forward-looking rational expectations models assume that students at time *t* are attempting, with some success, to predict wages and employment levels that will be in effect at the time they enter the job market, time *t+d*.

Following Ryoo and Rosen (2004), we control for supply shifters such as exogenous year-to-year changes in cohort size – which could affect the number of students completing college and who thus are eligible to enter PhD programs – and for wages and availability of students' alternative employment options by estimating a *relative* supply equation [20]. Formally, we begin by estimating the supply of new entrants into biomedical sciences graduate programs, as follows:
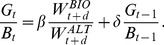
(1)


In this specification, the dependent variable represents the share of students who graduated with bachelor's degrees in biology or chemistry in year *t*, *B_t_*, who go on to enter graduate programs in biomedical sciences fields in the following academic year, *G_t_*.

Equation (1) asserts that the number of students entering biomedical sciences graduate programs in a given year should be determined by expected salaries for completed biomedical sciences PhDs *d* years hence, 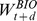, where *d* is the time delay between admission and completion. In addition to considering their expected future wages if they complete a biomedical sciences PhD, prospective students should also consider the opportunity cost of choosing to attend graduate school, instead of pursuing some alternative career path accessible to those who have earned only a bachelor's degree. For simplicity, in equation (1) we ignore the opportunity cost associated with years spent in graduate school, including only the expected (average) salary for those alternative careers *d* years hence, 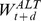.

Due to data limitations, we are unable to estimate the present value of prospective PhD students' expected lifetime earnings profiles directly. However, if we assume the wage profile by age and experience remains reasonably stable for biomedical scientists versus alternative careers over our relatively short 12-year period of analysis, this simplification – using relative average wages at students' expected year of graduation – should not influence our results. In their similar analysis of the market for engineers using a 40-year time series, Ryoo and Rosen (2004) found very little change in cross-sectional age-earnings profiles over time, and concluded that changes in relative wage levels dominated changes in the imputed discounted present value of graduates' lifetime earnings [20]. Our first explanatory variable thus represents financial prospects at graduation for a student who completes his or her biomedical sciences PhD in year 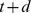, relative to average salaries in biology and chemistry majors' alternative career paths.

If graduate students have rational expectations regarding future market conditions, then students' expectations at time *t* for 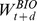 and 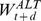 would, on average, equal the true values of each variable at time 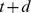. However, rational prospective students may also consider strategic responses by other prospective students (as well as current PhD scientists already participating in the labor market) to future changes in wages. To assess the salience of jobs growth for prospective biomedical scientists, and also to control for economy-wide employment shocks and demographically-driven retirement rates, we include an additional constructed variable calculated as the ratio of 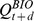 – a measure of the stock of biomedical scientists employed at time *t+d –* to *Q_t+d_* – total employment in S&E fields at time *t+d*:
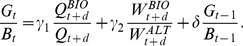
(2)


If students have “cobweb” expectations, then equation (2) is simplified. Students' expectations for 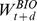 in equation (1) would simply be the current salary for biomedical scientists, 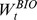, and their expectation for 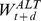
in equation (1) would be 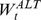. Likewise, students' expectations regarding future job growth in biomedical sciences occupations may depend only on current changes in employment levels for biomedical scientists, 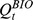 and current employment trends in other S&E fields, 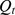. We test these alternative assumptions about students' expectations empirically.

Ignoring any additional information students might obtain during their years of graduate training that could update their expectations of their future earnings, the resulting change in labor supplied at time *t*, measured as the number of graduating biomedical sciences PhDs entering the labor market at time, *C_t_*, is primarily determined by *G_t-d_*, the number of students who entering graduate programs *d* years prior. The ratio of these two quantities, setting *d* = 6, provides us with a measure of six-year PhD completions, conditional on graduate student enrollments. With PhD completions thus calculated as our dependent variable, we then explore how changes in the proportion of full-time graduate students funded by NIH research assistantships, traineeships, or fellowships impact the quantity of biomedical scientists' labor supplied.

We estimate:
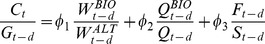
(3)where *F_t-d_* is the number of full-time biomedical sciences graduate students primarily supported by NIH funding sources, including research assistantships, traineeships and fellowships, and where *S_t-d_* represents the total number of graduate students enrolled, both at time *t-d*.

In our empirical analysis, we also investigated effects of changes in relative wages and employment at the time of PhD completion, *t*, as well as changes in the estimate of *φ*_3_ depending on the timing of changes in the proportion of students with NIH funding. For example, in addition to evaluating effects on PhD completions of changes in the NIH-funded fraction of students at time of their notional entry into the graduate programs, *t*–6, we also considered effects of changes in availability of NIH funding support in the intervening years between enrollment and completion, years *t*–5 through *t*.

### Modeling Demand for Biomedical Scientists

In recent years approximately 70% of new PhDs in biomedical sciences have taken postdoctoral research/training positions after graduation [8]. Many of these postdoctoral positions are funded by NIH extramural research and training grants, but some are in industry (e.g., at biopharmaceutical firms) or in government. To reflect both of these sources of labor demand, we represent the inverse demand function for biomedical scientists as follows:
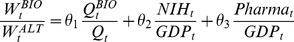
(4)


In our empirical analysis, following equation (4), the dependent variable is the log relative wage for biomedical scientists. The demand-shifters are total NIH obligations for R&D in year *t*, *NIH_t_*, representing demand for postdoctoral workers in academia and government, and estimated annual pharmaceutical and biotechnology industry R&D expenditures, *Pharma_t_*, to represent demand in industry. As noted above, both of these are expressed as logged ratios, with U.S. GDP in the denominators to control for macroeconomic trends. Theory predicts the sign on *θ_1_* will be negative, reflecting that an increase in labor market supply will, all else equal, reduce PhDs' market wage. Finally, for completeness, we also estimate the relative demand function directly, with quantity demanded as the dependent variable:
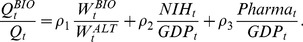
(5)


If an exogenous shock to wages or employment in one period affects unobserved factors in later periods, there may exist autocorrelation in the error terms. To assess this concern, we employed Durbin's [29] alternative test which permits lagged dependent variables, and also assesses presence of higher-order autocorrelation. Results from this test informed our choice in each case, whether to use first-order autoregressive (AR1) models that include the first lag of the dependent variable, as in equations (1) and (2), or first-difference the estimation equation, which removes the lagged dependent variable as in equation (3).

### Instrumental Variables (IV) Estimation

Structural market models, such as the supply and demand equations we estimate here, are characterized by jointly (simultaneously) determined prices and quantities. If these equations are estimated independently, without taking into account the information provided by other equations in the system, the regressions can yield biased results.

One established econometric approach for estimating simultaneous equations is two-stage least squares (2SLS) IV [24]. To implement 2SLS IV estimation for the labor supply equation, we need one or more instruments that are highly correlated with biomedical scientists' wages or with changes in the quantity of jobs available, but are otherwise uncorrelated with unobserved factors affecting the number of students enrolling in graduate programs or completing PhDs in the biomedical sciences. In their analysis of the market for engineers, Ryoo and Rosen (2004) employ the third and fourth lags of defense R&D spending as instruments, also using their ratio with total U.S. GDP, which they argue reflect changes in demand that affect the supply of bachelor's-level engineers *only* through their prospective future earnings [20]. For our analysis, one might presume that using lagged values of NIH R&D (again relative to U.S. GDP) would be analogous. However, because we find empirically that NIH R&D funding is in fact a strong *direct* predictor of labor supply as measured by graduate enrollments (Fig 5), it belongs in the supply equation as well as in the demand equation, and thus these measures cannot be used to resolve the system identification problem.

**Figure 7:**
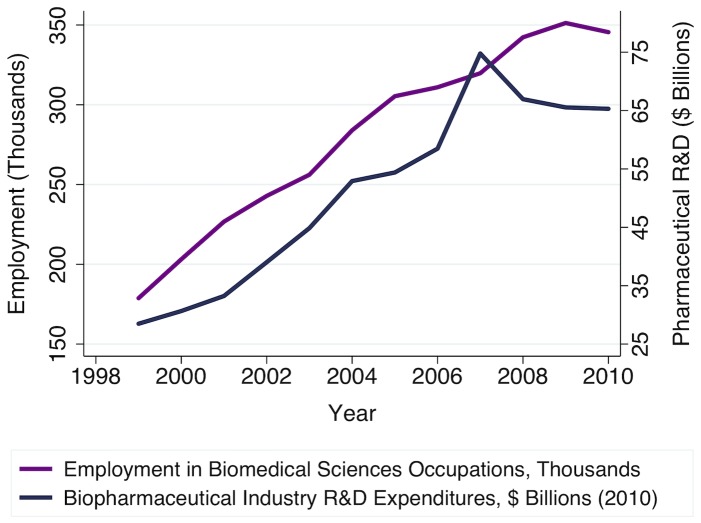
Biopharmaceutical industry R&D expenditures also drive changes in demand for biomedical scientists. Employment in biomedical sciences occupations, in thousands, is defined as in Figure 1, using BLS OES data. The biopharmaceutical industry R&D expenditures series shown is the composite Bureau of Economic Analysis pharmaceutical industry R&D satellite account series through 2007, linearly extrapolated based on Pharmaceutical Research and Manufacturers of America (PhRMA) members' R&D expenditures reported for years 2008 through 2010, in billions of constant (BRDPI-inflated) 2010 dollars.

Instead, in the empirical analyses that follow, we instrument for wages and job growth using the third and fourth lags of our two measures of private biopharmaceutical industry R&D expenditures, relative to GDP, as described above. The relevance of industry R&D expenditures to total biomedical sciences employment is visually apparent in Figure 7; however, we also provide a quantitative test of the relevance of our instruments, reporting the partial F-statistic for the excluded instruments from our first stage regressions.

For these lagged industry R&D expenditure variables to be valid instruments, they also must satisfy the IV exogeneity condition. That is, industry R&D expenditures can only be correlated with graduate student enrollments and PhD completions via the market price mechanism (wages) or changes in availability of jobs. At first blush, this seems reasonable: in FY2011, NSF's Survey of R&D Expenditures at Universities and Colleges found only 4.7% of academic life sciences research was funded by industry, compared to over 63% funded by Federal government sources. Similarly, FY2011 data from the NSF-NIH Survey of Graduate Students and Postdoctorates in Science and Engineering reveals that, for PhD-granting biosciences departments and programs, over 56% of full-time graduate students supported on research assistantships were funded by Federal agencies, but only 10% of research assistantships in these departments were funded by all external private sector sources combined, which includes industry funded R&D as well as nonprofit organizations (e.g., foundations).

In addition, although exogeneity of proposed instruments can never be definitively proven, when the number of plausibly exogenous instruments exceeds the number of problematic, endogenous explanatory variables, analysts can employ Hansen's overidentification test to assess whether the evidence supports exogeneity. Whenever possible, we therefore also report these overid test results for our IV models.

Finally, in principle the microdata sources we use here would permit calculation of standard errors for each of the survey-specific annual statistical estimates we derive and use in construction of our analytic dataset. However, when combining multiple microdata-based estimates, variation in precision due to differences in sample size across datasets, as well as within a given variable over time, can make statistical inference problematic, if one relies on the traditional least-squares assumption of constant variance. In the analysis that follows, we begin by treating the year, itself, as the unit of observation, and estimate survey-weighted averages and sums using microdata for each year. Because the individual surveys themselves are either population (census) surveys, or nationally representative samples, there is no reason to suspect these microdata would generate biased estimates of means or related linear combinations. Then, we present results for all models with their statistical significance tests based on corrected standard errors, robust to arbitrary heteroskedasticity.

### Models of the Demand for Biomedical Scientists

To provide additional support for our use of industry R&D expenditures as instruments, and also to explore the relative importance of NIH versus industry R&D funding in driving demand for biomedical scientists, we begin by estimating the market demand equations. Table 4 presents results from the demand side of the labor market. Whereas growth in the NIH budget drove year-to-year changes in biomedical sciences labor market conditions during the budget doubling period, since 2006 changes in industry R&D expenditures have become a more significant predictor of changes in market demand.

**Table 4:** Determinants of demand for biomedical sciences labor in the post-NIH-doubling environment, 2004–2010.

	Inverse Demand	“Regular” Demand Function
Change in Employment, t	−0.750***	
	(0.0833)	
Relative Wage, t		−1.251***
		(0.0624)
NIH R&D Obligations, t	0.717***	0.966***
	(0.0950)	(0.0540)
Industry R&D, t-1	0.200***	0.273***
	(0.0281)	(0.0124)
First-Stage F-statistic	27.60	701.7
Hansen's J-statistic	3.151	2.477
Hansen's J p-value	0.207	0.290

Standard errors robust to arbitrary heteroskedasticity are presented in parentheses below each coefficient estimate.

Both models above were estimated using seven years of statistical (microdata-based) and macroeconomic time series variables, years ranging from 2004 through 2010. The outcome variable for the inverse demand model in column (1) is the first-difference of the log ratio of weighted average salary for biomedical sciences occupations, versus that for alternative S&E careers. The outcome variable for model (2) is the first-difference of the log ratio of biomedical sciences employment to total employment in S&E bachelor's-degree fields. All explanatory variables in equations (1) and (2) are also first-differenced log ratios, with NIH R&D obligations and biopharmaceutical industry R&D expenditures (BEA composite measure) divided by U.S. GDP. Both models employ as instrumental variables the second through fourth first-differenced lags of the logged ratios of biopharmaceutical industry R&D expenditures (PhRMA measure) to U.S. GDP.

Both inverse and “regular” demand function formulations demonstrate significant negative relationships between wages and employment (i.e., quantity of labor demanded), as predicted by economic theory. The inverse demand function in column (1) indicates that a 10% increase in labor supply yields a 7.5% decrease in the market wage, all else equal. The regular demand function, which directly estimates elasticity of the labor quantity demanded to changes in market wages, finds demand is approximately unit elastic. That is, a 1% increase in wages would yield a 1.2% decline in demand for labor. In addition, we find that both NIH R&D obligations and biopharmaceutical industry R&D expenditures are significant demand shifters. Both of the models we estimated also indicate that changes in NIH funding have an approximately threefold higher effect on demand for biomedical scientists than similar-magnitude changes in industry R&D.

### Models of the Supply of Biomedical Scientists

Next, we assess whether students' enrollment in biomedical sciences graduate programs responds more to changes in current market conditions at time of entry, or alternatively whether enrollment trends appear to reflect rational expectations of future market conditions. Table 5 presents results from instrumental variables estimation of dynamic, first-order autoregressive (AR(1)) models, corresponding to equations (1) and (2) above, as well as from first-differenced equations that include the share of students with NIH graduate funding as an explanatory variable. As noted above, these different empirical specifications were chosen in part to mitigate autocorrelation in the errors, which we observed in some OLS versions of the models using Durbin's alternative test [29].

**Table 5:** Comparison of cobweb and rational expectations models of PhD student enrollments.

	Cobweb Expectations			Forward-Looking, Rational Expectations	
	(1)	(2)	(3)	(4)	(5)
Relative Wage	3.355**	2.864	3.857**	−0.324	−0.492
	(1.640)	(2.993)	(1.771)	(0.334)	(1.303)
Change in Relative Employment		0.530	0.222	0.305	−0.0179
		(0.559)	(0.195)	(0.275)	(0.190)
Wages & Employment Joint Significance, p-value		**<.001**	**0.038**	0.438	0.928
Percent of Students			0.853***		0.848***
with NIH Funding at Enrollment			(0.156)		(0.198)
First Lag, Relative Enrollment	1.551***	1.487***		1.019***	
	(0.258)	(0.464)		(0.0369)	
First-Stage F-statistic	23.71	65.72	2.227	6.914	4.747
Hansen's J-statistic	3.106	1.133	2.222	2.480	0.289
Hansen's J p-value	0.376	0.567	0.136	0.289	0.591
Observation-Years	8	8	8	8	8

*** p<0.01, ** p<0.05, * p<0.1.

Standard errors robust to arbitrary heteroskedasticity are presented in parentheses below each coefficient estimate.

All results presented are from instrumental variables estimation, with outcome variable the log of the ratio of first-time, full-time enrollment in U.S. biomedical sciences graduate programs each year to bachelor's degrees in biological sciences and chemistry-related fields from U.S. institutions in the previous academic year. Models (1), (2), and (4) are dynamic first-order autoregressive (AR(1)) models, whereas models (3) and (5) are instead first-differenced to remove autocorrelation. Models (1), (2), and (3) correspond to cobweb expectations, with all explanatory variables measured at time of students' entry into graduate programs. Models (4) and (5) assess rational or forward-looking expectations, with the relative wage and employment variables measured six years after entry into the graduate program. We instrument for wages and employment with third and fourth lags of each of our two measures of pharmaceutical industry R&D expenditures, divided by the U.S. Gross Domestic Product.

Columns (1) through (3) in Table 5 reflect the cobweb expectations assumption, wherein current market conditions are perhaps taken as a proxy for future earnings potential. In column (1), we find that a 1% increase in contemporaneous relative wages for biomedical sciences occupations versus alternative career fields yields an approximate 3.4% increase in graduate student enrollment (p<.05). Contemporaneous growth in the number of biomedical scientist jobs also significantly predicts increased entry into graduate programs (results not shown), with a 1% increase in jobs growth yielding near-proportional increase (0.9, p<.01) in graduate student enrollments. When we estimate the cobweb expectations model including both relative wages and changes in relative employment as in equation (2), we find similar results. Table 5 column (2) shows the effect on enrollments decomposed into a 2.9% increase in enrollments given a 1% increase in wages, and a 0.53% increase in enrollments with a 1% increase in biomedical scientist jobs. Although these individual effects are imprecisely estimated, a partial F-test reveals that taken together, market conditions at time of enrollment (i.e., current wages and employment) are jointly highly significant (p<.001).

Table 5 column (3) shows that graduate student enrollments remain highly responsive to current changes in relative wages, even after we control for changes in availability of NIH funding for students. We estimate a 1% increase in biomedical scientists' current relative wage yields nearly a 3.9% increase in new enrollments, holding the NIH-funded share of biomedical sciences graduate students constant. However, we also find that, controlling for concurrent changes in market wages, increased availability of NIH support is still a significant positive predictor of graduate student enrollments. Each 1% increase in the number of full-time biomedical sciences graduate students supported by the NIH yields an increase of 0.85% in first-time, full-time enrollments (p<.01). At the means, this indicates each additional student funded by NIH fellowships, traineeships, research assistantships, or other mechanisms of NIH support is associated with approximately one additional student (estimate at means = 0.99) entering full-time graduate training that same year.

In contrast with this evidence of significant support for the cobweb expectations assumption, columns (4) and (5) in Table 5 provide no evidence of forward-looking, rational expectations as a driver of graduate student enrollments. Specifically, we find no significant relationships between students' entry into graduate programs and either the relative wage for biomedical scientists, or the relative growth in job opportunities realized six years later. However, our point estimate of the effect of current changes in the NIH-funded percentage of full-time students on graduate student enrollments remains nearly identical and highly significant (0.85, p<.001).

Next, we examine the interaction of PhD completions, wages, and NIH funding levels. The dependent variable for the models presented in Table 6 is our proxy for the six-year completion rate described above, calculated as the first-differenced log ratio of PhDs earned in biomedical sciences programs each year to first-time enrollments in those graduate programs six years prior. Despite the significant enrollment response we found in Table 5 associated with contemporaneous increases in biomedical scientists' relative wages at time of entry, Table 6 column (1) shows no corresponding significant increase in PhD completions six years later. In contrast, to the extent that prospective graduate students were responding not to changes in biomedical scientists' relative wages, but rather to relative employment growth in biomedical scientist occupations, the latter does significantly predict relative increases in PhD completions six years later, with a 1% increase in relative job growth yielding a 0.57% increase in PhD completions.

**Table 6:** Effects of market conditions at time of enrollment on PhD completions.

	(1)	(2)	(3)
Relative Wage, t–6	2.088		−0.721
	(1.444)		(0.856)
Change in Employment, t–6	0.567**	0.873***	1.027***
	(0.240)	(0.0737)	(0.192)
Percent of Graduate Students		−1.931***	−1.915***
with NIH Support, t–6		(0.491)	(0.661)
First-Stage F-statistic	3.26	90.51	1.964
Hansen's J-statistic	–	1.38	–
Hansen's J p-value	–	0.24	–
Observation-Years	5	5	5

*** p<0.01, ** p<0.05, * p<0.1.

Standard errors robust to arbitrary heteroskedasticity are presented in parentheses below each coefficient estimate.

Results are from instrumental variables estimation, with dependent variable the first-differenced, logged ratio of PhD completions to first-time graduate student enrollment in U.S. biomedical sciences graduate programs six years prior. All explanatory variables are likewise measured six years prior, relative and first-differenced, and use the sixth lags of our two measures of relative industry R&D expenditures as instruments. As in Table 5, here the combination of first-differencing and use of lagged industry R&D estimates, for which data are only available 1998 onwards, limits the number of observation-years available (with their attendant lags) for time-series estimation.

Due to the apparent lack of predictive power we observed for entry-contemporaneous wages on completed PhDs, we exclude wages from the model presented in Table 6 column (2), and replace it with the proportion of full-time graduate students with NIH funding six years prior. Growth in biomedical sciences employment at time of first enrollment remains a significant and positive predictor of six-year PhD completions in this model (p<.001). However, whereas the overall effect of relative wages on PhD completions was insignificant, the effect of an increase in the share of students with NIH funding on PhD completions six years later is strongly negative. Controlling for the direct effects of NIH funding on prospective graduate students' decision to enroll, we find that a 1% increase in the share of students supported by NIH yields nearly a 2% *decrease* in completed PhDs six years later. Including all three (relative wages, job growth, and share of students with NIH funding) as in column (3) yields very similar results.

**Figure 8:**
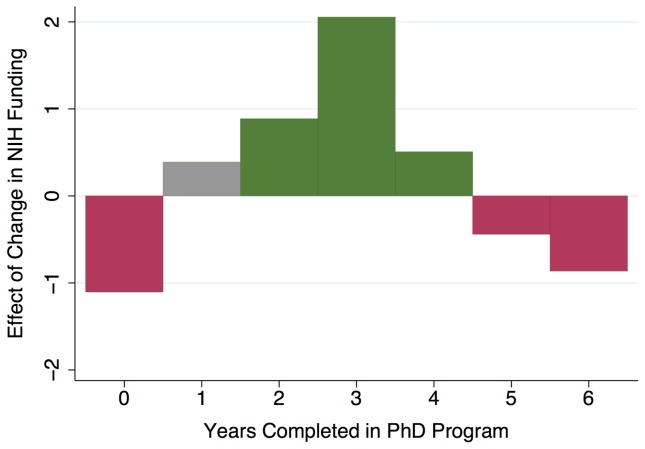
Timing of changes in availability of NIH-funded support impact PhD completions. Results are from econometric models estimating effects of a change in the percent of full-time graduate students with NIH funding support that occurs at time of students' entry, or in each of the six years thereafter. First-time, full-time enrollments and percent of graduate students with NIH funding calculated from the NSF Survey of Graduate Students and Postdoctorates in Science and Engineering, for departments and programs offering PhDs in biomedical sciences fields. PhD completions are from the Survey of Earned Doctorates – Doctorate Records File. Green bars indicate significant positive effects (p<.10); red bars indicate significant negative effects (p<.05).

Finally, Figure 8 provides a graphical depiction of our econometric results for a series of models estimating the effects of an increase in availability of NIH funding on six-year PhD completions, based on the notional timing of that change with respect to students' doctoral training. We again find a significant negative effect of increased availability of NIH funding on six-year completion rates when the increase in proportion of students funded occurs concurrently with students' entry into the doctoral program. Likewise, unsurprisingly, when more NIH funding becomes available for graduate students, the number of PhDs graduating that year declines. In contrast, when the proportion of students with NIH funding increases as students enter the third and fourth years of their program, the number of completed PhDs two to three years thereafter significantly improves.

## Discussion

Our results suggest that prospective graduate students are well-informed and responsive with respect to current market wages at time of enrollment, and thus may have cobweb expectations with respect to their future career prospects. Our estimate of the elasticity of supply for this market is approximately 3.4, consistent with previous results estimated on the market for engineers (range 2.5–4.5) [20]. Enrollments increase slightly less than proportionally with increased availability of NIH support for graduate students, suggesting little “crowding-out” of other funding sources occurs. However, although first-time graduate enrollments do appear to respond both to current relative wages for biomedical scientists and to availability of NIH funding for graduate students, neither of these correspond to increases in PhD completions six years later.

Successful PhD students seem to select into biomedical sciences training programs due to beliefs about their chances of securing a job as a biomedical scientist – that is, due to relative growth in employment – but seemingly without regard to the salaries paid in these fields. However, consistent with economic cobweb expectations models, these beliefs seem to depend on current market signals at the time of their enrollment, rather than anticipation of future market conditions. Unfortunately, in the post-doubling environment, the jobs these PhDs expected to fill have not materialized. Including the share of graduate students with NIH funding as an additional explanatory variable has little influence on these results.

On the other hand, we do find a significant negative impact on six-year PhD completions when the NIH-funded share of graduate students increases either at the time of students' first enrollment, or as students complete their fifth or sixth years of doctoral training. In the former case, our finding suggests that increases in universities' NIH R&D funding may cause them to admit students on the margin, who are less likely to complete PhDs. Due to how we construct this variable, this apparent decline in PhD completions after six years could also reflect increases in admissions and enrollments of Master's degree students at PhD-granting programs. Alternatively, perhaps prospective students who enroll in graduate programs in response to observed increases in biomedical scientists' wages are also more likely to perceive changes in labor market conditions while enroute to their degrees, and – due to declining job growth and early-career wages – they chose to drop out and pursue alternative careers.

For PhD students nearing the six-year mark, it seems that increasing availability of NIH support may make it less likely that they complete their degrees on time. This may provide some evidence in favor of capping individual students' total years of NIH support, as recommended by the NIH ACD task force report [8].

Our analysis also shows that demand for biomedical scientists is responsive to changes in wages, with increases in wages yielding a near-proportional decrease in the quantity of jobs for biomedical sciences occupations. This result suggests that policies mandating higher wages in this market – for example, exogenous increases in the NIH postdoctoral stipend schedule, holding total NIH R&D funding levels constant – may have unintended consequences in decreasing the number of jobs available for new PhDs.

As noted above, due to constraints in the availability and comparability of wage and employment data prior to 1999, our study focuses on graduate students entering or graduating from PhD programs from 1999 onwards. This was clearly a period of dramatic change in the biomedical sciences research funding environment, and that in itself makes our study period one of particular policy (and scholarly) interest. However, due to these data constraints, we are unable to make any inferences about earlier generations of biomedical sciences PhDs and their responsiveness to market signals, nor any comparisons between the behaviors we observe during this period and previous market behaviors.

Qualitative research with PhD-granting departments and with students who exit prior to earning their PhDs may allow us to parse the relative contributions of possible declining aptitude or inclination towards research careers among admitted students, versus increased faculty incentives to delay students' graduation, versus growth in attractive job opportunities for students who leave their programs without completing their PhDs. Any of these could potentially explain the lower six-year completions we observe. In any case, it is clear that labor supply in the market for biomedical scientists is highly responsive to changes in NIH funding, due to its effects both on financial support for students and on availability of jobs. Dynamic modeling of the feedback and interactions in this system will be critical to informing future NIH policy changes, for a more sustainable biomedical research workforce.

## References

[1] Garrison HH, Stith AL, Gerbi SA (2005) Foreign postdocs: the changing face of biomedical science in the US. FASEB J 19: 1938–1942. 16319136 10.1096/fj.05-1203ufm

[2] Stephan P (2012) Perverse incentives. Nature 484: 29–31. 22481339 10.1038/484029a

[3] Levitt DG (2010) Careers of an elite cohort of U.S. basic life science postdoctoral fellows and the influence of their mentor's citation record. BMC Med Educ 10: 80. 21078180 10.1186/1472-6920-10-80 PMC2996387

[4] Bedard K, Herman DA (2008) Who goes to graduate/professional school? The importance of economic fluctuations, undergraduate field, and ability. Econ Educ Rev 27: 197–210.

[5] Loscalzo J (2006) The NIH budget and the future of biomedical research. N Engl J Med 354: 1665–1667. 16625006 10.1056/NEJMp068050

[6] National Research Council (2011) Research-Doctorate Programs in the Biomedical Sciences: Selected Findings from the NRC Assessment; Lorden JF, Kuh CV, Voytuk JA, editors. Washington, DC: National Academies Press. 22259822

[7] Teitelbaum MS (2008) Research funding – Structural disequilibria in biomedical research. Science 321: 644–645. 18669847 10.1126/science.1160272

[8] Tilghman S, Rockey S, Degen S, Forese L, Ginther D, et al.. (2012) Biomedical Research Workforce Working Group Report. Bethesda, MD: National Institutes of Health.

[9] Stephan P (2012) How Economics Shapes Science: Harvard University Press.

[10] Mullen AL, Goyette KA, Soares JA (2003) Who goes to graduate school? Social and academic correlates of educational continuation after college. Sociol Educ 76: 143–169.

[11] Brotman JS, Moore FM (2008) Girls and science: a review of four themes in the science education literature. J Res Sci Teach 45: 971–1002.

[12] St John EP, Paulsen MB, Carter DF (2005) Diversity, college costs, and postsecondary opportunity: an examination of the financial nexus between college choice and persistence for African Americans and Whites. J Higher Educ 76: 545–569.

[13] Eagan MK, Newman CB (2010) Investing in human capital: underrepresented racial minorities' intentions to attend graduate school in STEM fields. Los Angeles, CA: UCLA Higher Education Research Institute. Available: http://heri.ucla.edu/nih/downloads/Eagan Newman – Investing in Human Capital – AERA 2010.pdf. Accessed: 2013 May 15.

[14] Roach M, Sauermann H (2010) A taste for science? PhD scientists' academic orientation and self-selection into research careers in industry. Res Policy 39: 424–434.

[15] Whittington KB, Smith-Doerr L (2008) Women inventors in context: Disparities in patenting across academia and industry. Gend Soc 22: 194–218.

[16] Husbands Fealing K, Myers SL (2012) Pathways v. pipelines to broadening participation in the STEM workforce. University of Minnesota, via SSRN website. Available: http://dx.doi.org/10.2139/ssrn.2020504. Accessed 2013 Nov 18.

[17] Greene J, Stockard J, Lewis P, Richmond G (2010) Is the academic climate chilly? The views of women academic chemists. J Chem Educ 87: 381–385.

[18] Sauermann H, Roach M (2012) Science PhD career preferences: Levels, changes, and advisor encouragement. PLoS One 7 (5): e36307 doi:10.1371/journal.pone.0036307 10.1371/journal.pone.0036307 PMC3342243 22567149

[19] Taylor JB (1985) Rational expectations models in macroeconomics. In: Arrow KJ, Honkapohja S, editors. Frontiers of Economics. New York: Basil Blackwell.

[20] Ryoo J, Rosen S (2004) The engineering labor market. J Polit Econ 112: S110–S140.

[21] Freeman RB (1976) A cobweb model of the supply and starting salary of new engineers. Ind Labor Relat Rev: 236–248.

[22] Blume-Kohout ME, Adhikari D (2012) Training the biomedical workforce: Does funding mechanism matter? Southern Economic Association 82nd Annual Meeting New Orleans, LA, 2012 November 16–18.

[23] Ehrenberg RG, Rees DI, Brewer DJ (1993) Institutional responses to increased external support for graduate students. Rev Econ Stat 75: 671–682.

[24] Angrist JD, Krueger AB (2001) Instrumental variables and the search for identification: From supply and demand to natural experiments. J Econ Perspect 15: 69–85.

[25] Bureau of Labor Statistics, U.S. Department of Labor Occupational Employment Statistics. Available: http://www.bls.gov/oes/tables.htm. Accessed 2011 April 6. Accessed 2012 Oct 26.

[26] Bureau of Labor Statistics, U.S. Department of Labor (2007) Technical Notes for 1999 OES Estimates. Available: http://www.bls.gov/oes/1999/oestec99.htm. Last modified: 2007 January 10. Accessed 2013 Nov 18.

[27] Fox MF, Stephan PE (2001) Careers of young scientists: preferences, prospects and realities by gender and field. Soc Stud Sci 31: 109–122.

[28] U.S. Bureau of Economic Analysis (2010) Table C. Contributions to the Annual Growth Rate of Real Business Sector Investment in R&D, 1999–2007. Research and Development Satellite Account. Available: http://bea.gov/newsreleases/general/rd/2010/xls/R&DSA_2010.xls. Accessed: 2012 Oct 26.

[29] Durbin J (1970) Testing for serial correlation in least-squares regression when some of the regressors are lagged dependent variables. Econometrica: 410–421.

